# Expanding the Clinical Spectrum of Sotos Syndrome in a Patient with the New “c.[5867T>A]+[=]”; “p.[Leu1956Gln]+[=]” *NSD1* Missense Mutation and Complex Skin Hamartoma

**DOI:** 10.3390/ijms19103189

**Published:** 2018-10-16

**Authors:** Annalisa Mencarelli, Paolo Prontera, Amedea Mencarelli, Daniela Rogaia, Gabriela Stangoni, Massimiliano Cecconi, Susanna Esposito

**Affiliations:** 1 Pediatric Clinic, Department of Surgical and Biomedical Sciences, Università degli Studi di Perugia, Piazza Menghini 1, 06129 Perugia, Italy; almiomedico@gmail.com; 2 Medical Genetics Unit, S. Maria della Misericordia Hospital, Piazza Menghini 1, 06129 Perugia, Italy; paolo.prontera@ospedale.perugia.it (P.P.); amedea.mencarelli@unipg.it (A.M.); daniela.rogaia@unipg.it (D.R.); gabriela.stangoni@ospedale.perugia.it (G.S.); 3 Laboratory of Human Genetics, Galliera Hospital, Mura delle Cappuccine, 14, 16128 Genoa, Italy; massimiliano.cecconi@galliera.it

**Keywords:** missense mutation, *NSD1* gene, overgrowth, skin hamartoma, Sotos syndrome

## Abstract

Sotos syndrome is one of the most common overgrowth diseases and it predisposes patients to cancer, generally in childhood. The prevalence of this genetic disorder is 1:10,000–1:50,000, and it is characterized by wide allelic heterogeneity, with more than 100 different known mutations in the nuclear receptor-binding SET domain containing protein 1 (*NSD1*) gene. Most of these alterations are deletions and common micro-deletions with haploinsufficiency. Singular variants are missense mutations. The present study reports a case of a 4-year-old boy with specific clinical features of Sotos syndrome and a particular complex skin hamartoma on the right femoral side, in addition to other minor findings, such as a “café-au-lait” spot on the right hemithorax and syndactyly of the second and third right toes. *NSD1* gene analysis identified a de novo missense mutation, “c.[5867T>A]+[=]”; “p.[Leu1956Gln]+[=]”, that was not previously described in the literature. This mutation was localized to the functional domain of the gene and was likely the cause of Sotos syndrome in our patient. We also compared aspects of our patient’s condition with the clinical features of tuberous sclerosis (TSC), which is an autosomal neurocutaneous syndrome caused by mutations in the *TSC1/TSC2* genes. These genes control cell growth and cell survival. This disorder is characterized by hamartomas in multiple organ systems, several coetaneous abnormalities, epilepsy, and increased risk of several types of tumors.

## 1. Introduction

Somatic growth is an increase in cell size and number, whereas overgrowth is a condition that is characterized by an extreme physical size, learning disability, and overgrowth of tissues [1]. Sotos syndrome (# MIM 117550, for those forms being associated with nuclear receptor-binding SET domain containing protein 1 (*NSD1*) gene mutations) is a childhood overgrowth syndrome. It is usually a dominant disorder, or rarely a de novo disorder, and is associated with pre- and post-natal growth, advanced bone age, macrocephaly, peculiar facial features with a large skull and pointed chin, brain anomalies (cerebral gigantism), developmental delay with or without seizures, and a high risk of benign/malignant cancer [2,3].

Sotos syndrome is caused by a wide spectrum of molecular genetic disorders that result in haploinsufficiency of the *NSD1* at chromosome 5q35. *NSD1* belongs to a family of nuclear receptors and plays a role in cell growth and differentiation by functioning as a transcriptional repressor or transcriptional activator [4]. More than 400 mutations in the *NSD1* gene are reported in the Human Gene Mutation Database (HGMD) and in the Leiden Open Variation database (associated with Sotos syndrome. Of these mutations, about 35% are small or gross gene deletions, ~25% are missense variants, ~20% are nonsense variants, ~15% are small insertions, ~5% are splice variants, and the remaining 5% are complex rearrangements. Clinical manifestations of Sotos syndrome include feeding difficulties, variable types of renal and cardiac anomalies, seizures, scoliosis, and mental retardation [5,6]. The risk of developing neoplasms in Sotos syndrome is approximately 2–7% [7,8]. Sotos syndrome with *NSD1* mutation is associated with several types of cancer, including neural crest tumors, sacrococcygeal teratomas, Wilms tumors, neuroblastoma, leukemia and hepatocellular carcinoma [9,10]. Phenotypical differences are reported between patients with deletions or missense/nonsense mutations in the *NSD1* gene, which demonstrates an evident genotype-phenotype correlation in Sotos syndrome. Patients with micro-deletions exhibit more severe learning difficulties and behavioral problems and fewer somatic overgrowths than patients with missense/nonsense mutations [11,12].

The present study describes a case of peculiar complex skin hamartoma that developed in a child with Sotos syndrome that was associated with a de novo missense mutation in the *NSD1* gene. We also compared this disorder with tuberous sclerosis, which is a neurocutaneous syndrome that is characterized by hamartomas in multiple organ systems.

### Case Report

**Figure 1:**
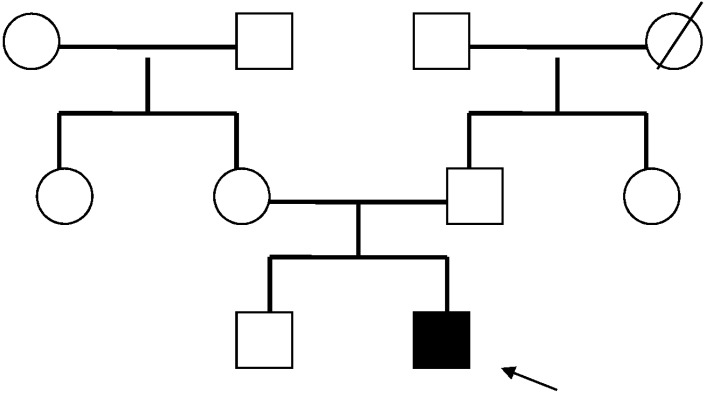
The pedigree diagram of our patient. The black arrow indicates the propositus.

Our patient was a 4-year-old Italian boy who visited our pediatric hospital for incidental head trauma with a linear fracture of the right parietal bone and a small hematoma. He presented with a Glasgow Coma Scale of 15/15 and normal neurological testing. His weight was 21 kg (>97th percentile), height was 113 cm (>97th percentile), and head circumference was 54.5 cm (>97th percentile). The patient exhibited dysmorphic facial appearance with trigonocephaly, frontal bossing, large ears, prominent chin, and high palate with dental malposition. Physical examination of the skin found one “café-au-lait” spot on the right hemithorax (2 cm × 0.5 cm) and an irregular skin lesion with a rubbery consistency and fibrosis (3 cm × 4 cm) on the right femoral side. He also exhibited syndactyly of the second and third right toes and learning difficulties. He was born at full-term via caesarean section because of macrosomia. His birth weight was 4.050 g (>97th percentile). His parents were healthy and unrelated. His older brother was also healthy and had normal development. His grandfather died at the age of sixty to chronic lung disease (Figure 1).

## 2. Materials and Methods

### 2.1. Laboratory and Instrumental Analyses

**Figure 2:**
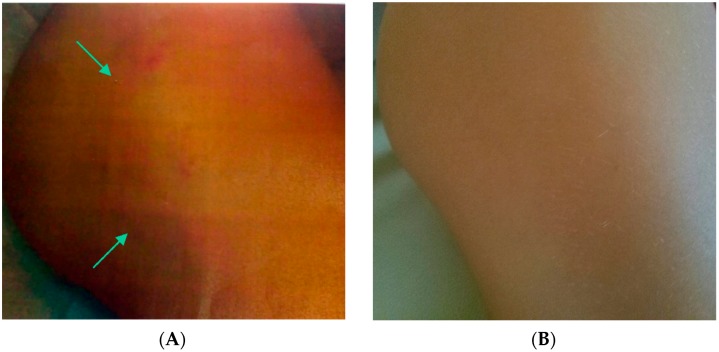
Video dermoscopy examination. (**A**) Complex skin hamartoma on the right femoral side of the patient. The image shows a wide fibrous and hyperemic hamartoma, and the green arrows indicate the skin lesion. (**B**) The skin of the right femoral side of a healthy control.

Initial investigations included a blood cell count (white blood cells 4600/µL; red blood cells 3,750,000/µL; Hb 13.8 g/dL; platelets 350,000/µL), liver function tests (SGOT 20 U/L, SGPT 25 U/L), kidney function tests (creatinine 0.6 mg/dL, azotemia 20 mg/dL), serum electrolyte concentration (Na 136 mEq/L; K 4.5 mEq/L; Cl 101 mEq/L; Ca 5.30 mEq/L), thyroid function (TSH 3.0 mIU/L) and tumoral markers (alpha-fetoprotein, carcinoembryonic antigen, and beta-human chorionic gonadotropin), which exhibited normal results. The bone age of our patient was advanced; the left hand and wrist radiograph estimated a bone age of 5.5 years. The standard deviation score was +2.8 (>2 standard deviations; +1.88 represented the 97th percentile and −1.88 the 3rd percentile). An echocardiogram revealed a normal aortic diameter with no evidence of mitral valve prolapse. Electroencephalography demonstrated a regular and symmetrical organization of background activity. Brainstem response electric audiometry was normal bilaterally. The abdomen ultrasonography was normal, and brain magnetic resonance imaging revealed left choroid cysts, mild dilatation of the perivascular spaces of Virchow-Robin, and hypoplasia of the corpus callosum. Dermatological and histopathological tests of the skin lesion on the right femoral side primarily revealed tissue with dilated follicles, basophilic cells, and concentric fibrosis, consistent with the diagnosis of complex skin hamartoma (Figure 2).

Conventional karyotyping revealed a normal male with 46, XY. A DNA screen for FRAXA Syndrome and the MLPA (multiplex ligation-dependent probe amplification) genetic examination were negative.

The presence of these peculiar clinical features was consistent with the suspected Sotos syndrome.

### 2.2. Ethics Committee Approval

Clinical information and blood samples were obtained after approval from the Ethics Committee of the Umbria Region with signed informed consent by both parents. DNA was extracted from peripheral blood leukocytes of the proband using a QIA Symphony SP robot (Qiagen, Hilden, Germany) according to the manufacturer’s protocol. High-quality DNA was quantified using a Quantifluor Fluorometer (Promega, Madison, WI, USA).

### 2.3. Genetic Analyses

**Figure 3:**
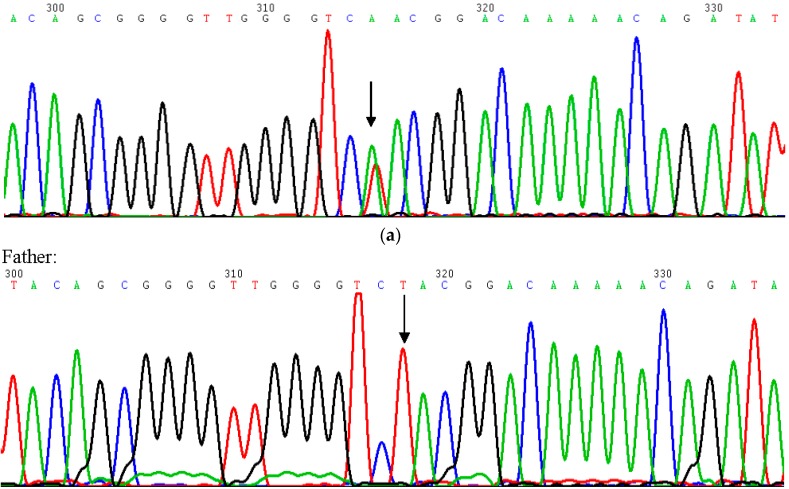

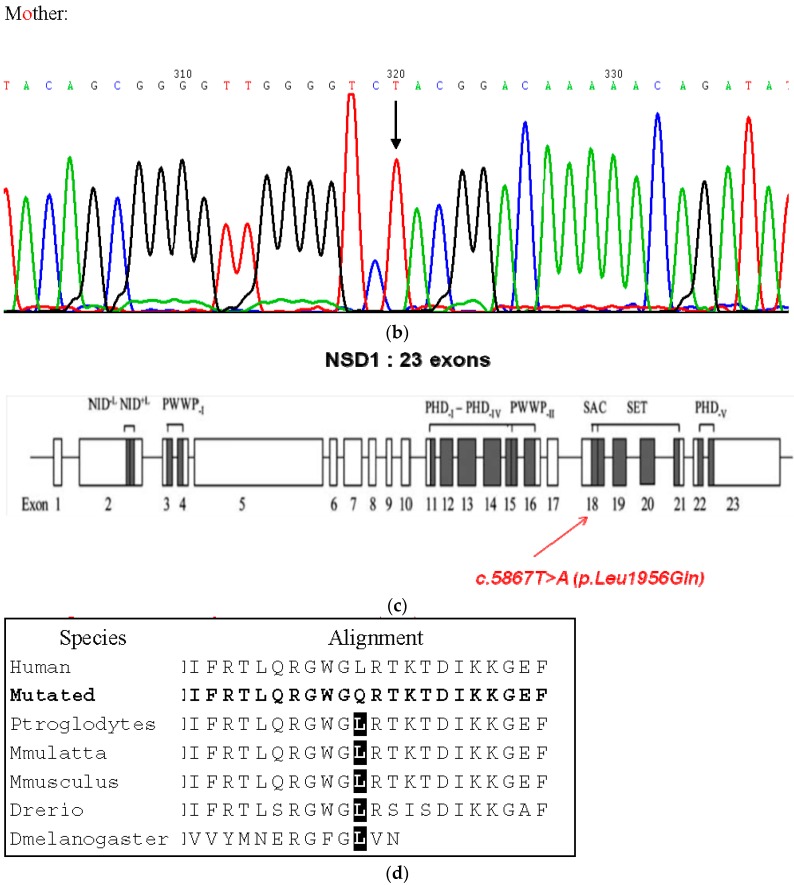
(**a**) A partial electropherogram of the Sanger sequencing of *NSD1* in the patient shows (black arrow) the “c.[5867T>A]+[=]”; “p.[Leu 1956Gln]+[=]”. Note the green peak, indicating the presence of Adenine, overlapping the red peak, indicating the presence of the wild-type Thymine, which identifies the heterozygous condition. (**b**) A partial electropherogram of the Sanger sequencing of NSD1 in the parents of the patient. Analysis of parental DNA (black arrows) defined the de novo origin of the missense mutation. (**c**) The exonic structure of *NSD1* (nuclear receptor SET domain containing protein-1) with the functional domains shaded. The red arrow indicates the de novo point mutation in exon 18: “c.[5867T>A]+[=]”; “p.[Leu1956Gln]+[=]”. (**d**) Conservation of the amino acid Leucine (L) among different species. “Human” refers to the wild-type amino acid sequence in humans. “Mutated” refers to the missense mutation of our patient, where Q is for Glutamine (Gln).

Genomic DNA was extracted from the peripheral blood of the patient and parents using standard protocols and the Qiagen Puregene Blood Core Kit. We examined all of the exons of the *NSD1* gene using denaturing high-performance liquid chromatography, and sequences of chromatograms were compared to those from normal control samples and reference sequences for the *NSD1* gene (Figure 3a,b) [13].

A heterozygous “c.[5867T>A]+[=]”; “p.[Leu1956Gln]+[=]” variant was identified in exon 18 of the *NSD1* gene (GenBank Accession: NM_022455.4) (Figure 3c,d). The variant was of de novo origin because it was not detected in the parents [13]. This missense mutation was not previously described in the literature and was localized to the functional domain of the *NSD1* gene. This missense mutation was likely the cause of Sotos syndrome in this patient.

## 3. Discussion

The case examined in this study exhibited a de novo heterozygous missense mutation “c.[5867T>A]+[=]”; “p.[Leu1956Gln]+[=]” in the *NSD1* gene, which expands our knowledge of the spectrum of genotype-phenotypes associated with Sotos syndrome. The identified nucleotide variation involved an amino acid change that was not previously described in the literature. However, its pathogenicity is apparent because its origin was de novo and the substituted amino acid was in a functional domain of the protein. The “c.[5867T>A]+[=]”; “p.[Leu1956Gln]+[=]” mutation found in the exon 18 of the *NSD1* gene in our patient has not been previously reported. Another missense mutation has been reported at codon 1955 of the *NSD1* protein (p.Gly1955Asp) in a patient with Sotos Syndrome by Tatton-Brown et al. [12]. These authors demonstrated by statistical analysis that the domain distribution of the de novo missense mutations was significantly different compared to those of known polymorphisms. Other studies identified a clustering of missense mutations in the C-terminal half of NSD1, in highly conserved functional domains, encoded by exons from 13–23 [14]. These observations reinforce the assumption of a pathogenetic effect of the mutation identified in our patient that is located within the functional SET domain (amino acids 1942–2059). The SET domain belongs to the class V-like SAM-binding methyltransferase superfamily and plays an important role in chromatin-mediated regulation during development [15]. The amino acid sequence in a SET domain is highly conserved among species. This suggests that a single missense mutation in this region can modify the most relevant functions of the NSD1 protein (Figure 3d). One of the functions that might be lost is the ability to repress growth-promoting genes. This can produce a clinically relevant overgrowth phenotype. Our patient also exhibited the primary clinical features of Sotos syndrome, such as macrosomia, tall stature, macrocephaly, prominent chin, and developmental delay, further supporting the association between the specific mutation and genetic disease. Macrocephaly is a cardinal feature of Sotos syndrome [9] with the presence of other specific findings, including advanced bone age [16], learning difficulties, and brain malformations, such as hypoplasia of the corpus callosum and dilation and asymmetry of cerebral ventricles [17]. Sotos syndrome and other overgrowth diseases are associated with a risk of tumorigenesis, and several types of benign and malignant cancers have been observed. The male/female ratio is 2:1. The appearance of tumorigenesis in Sotos syndrome often occurs in childhood, in contrast with other overgrowth disorders [18].

Our patient also exhibited a complex skin hamartoma and skin macule, which are described in neurocutaneous disorders, such as tuberous sclerosis. Hamartoma is a benign skin tumor that exhibits distinctive histopathological features, including hair follicles, epidermal cysts, and an increased number of collagen fibers extending to subcutaneous tissue. This type of skin lesion was observed in our patient, and it is associated with other genetic syndromes that are characterized by abnormalities of the skin in most cases, such as tuberous sclerosis (TSC) [19]. TSC is an autosomal neurocutaneous syndrome that is associated with epilepsy, mental retardation, hamartomas in multiple organs (such as the skin, brain, eyes, heart, lungs, kidneys, and bowel), and several types of skin lesions. This disorder is caused by mutations in the *TSC1/TSC2* genes, which control cell growth and cell survival [20]. The primary features of TSC are hypomelanotic macules (“white spots”), which were absent in our patient, who presented with a “coffee-milk” spot, facial angiofibroma, multiple hamartomas, shagreen patches, ungula fibromas, cortical dysplasias, and increased risk of several types of tumors, including astrocytoma, cardiac rhabdomyoma, and angiomyolipomas [19,21]. The clinical features of TSC are compared with the features of Sotos syndrome (Table 1).

Our patient exhibited particular skin features and a new missense mutation in the *NSD1* gene. These data demonstrate that the *NSD1* gene is subjected to strong allelic heterogeneity. Previous studies identified 100 mutations of the *NSD1* gene [5,22]. Genotype-phenotype correlations are described in patients with Sotos syndrome. Patients with micro-deletions exhibit more severe learning difficulties and behavioral problems and fewer somatic overgrowths than patients with missense/nonsense mutations [11,12].

## 4. Conclusions

Sotos syndrome is a genetic disease caused by *NSD1* mutations or micro-deletions. In addition to the traditional characteristics of Sotos syndrome, our patient developed rare clinical manifestations, including a complex skin hamartoma and other minor findings, such as a “café-au-lait” spot on the right hemithorax and syndactyly of the second and third right toes. A heterozygous de novo “c.[5867T>A]+[=]”; “p.[Leu1956Gln]+[=]” variant was identified in exon 18 of the *NSD1* gene. This missense mutation was not previously described in the literature and was localized in the functional domain of the *NSD1* gene. Therefore, this mutation was likely the cause of Sotos syndrome in our patient. Knowledge of the primary overgrowth syndromes and use of genetic analyses to confirm the specific disorder are important to improve patient care and prevent complications and cancer predisposition.

## Figures and Tables

**Table 1:** Clinical features of tuberous sclerosis and Sotos syndrome.

Features	Sotos Syndrome	Tuberous Sclerosis
Somatic overgrowth	+++	−
Macrocephaly	++	−
Facial findings	++	−
Brain malformations	++	++
Seizures	+/−	+++
Increased tumorigenesis	++	++
Multiple Hamartomas	−	++
Hypomelanotic macules	−	+++
Learning disabilities	++	++

+++, very frequent; ++, frequent; +/−, rare; −, absent.
